# Randomized approaches for identifiability analysis and model reduction in fibrin polymerization kinetics

**DOI:** 10.1098/rsif.2026.0210

**Published:** 2026-11-04

**Authors:** Julia M. Sanger, Ana Sheridan-Munana, Ashley C. Brown, Mansoor A. Haider

**Affiliations:** ^1^ Department of Mathematics, North Carolina State University at Raleigh, Raleigh, NC, USA; ^2^ Joint Department of Biomedical Engineering, North Carolina State University at Raleigh, Raleigh, NC, USA; ^3^ Joint Department of Biomedical Engineering, The University of North Carolina at Chapel Hill, Chapel Hill, NC, USA

**Keywords:** wound healing, fibrin polymerization, identifiability analysis, parameter estimation, model reduction

## Abstract

In the early stages of wound healing, fibrin polymerization plays an essential role in clot formation and subsequent healing. Sialic acid is known to impact fibrin polymerization kinetics, but the exact mechanisms are not well understood. We build upon existing studies using a reaction system-based mathematical model for fibrin matrix formation by introducing randomization to local sensitivity-based parameter identifiability analysis, with biochemical reaction rates serving as the model parameters. The application of randomization to initial parameter values facilitates greater exploration throughout parameter space. Our approach generates several model realizations that are screened for the quality of curve fits to data for fibrin matrix concentration and then used to determine consistently unidentifiable parameters as candidates for removal. Among these candidate reduced models, the most accurate one is selected and then used to estimate and compare influential parameter values from data corresponding to three experimental designs with different levels of sialic acid. Using statistical significance tests on the estimated values of influential parameters, results are interpreted to inform the potential mechanisms that may be altered by sialic acid concentration on fibrinogen.

## 1. Introduction

Haemostasis is the first phase of wound healing and results in the initial formation of a fibrin clot. It is followed by three additional and overlapping phases: inflammation, proliferation and remodelling [1]. Vascular injury causes endothelial cell damage, after which platelet activation and fibrin clot formation ensue through polymerization of fibrin. Fibrin clot formation is mediated by the enzyme thrombin and involves a cooperative mechanism with multiple intermediate complexes [2]. A variety of mathematical modelling approaches have been used to study one (or a few) stages of wound healing including fibrin polymerization [3–12]; see also [13–16] for reviews.

Biochemical reaction kinetics models are commonly used to study haemostasis, especially when measurements of fibrin matrix content over time are available but not spatially delineated. In such data-driven studies, it is also commonly the case that data are available for only a small number of species in the biochemical reaction system. This poses potential challenges for parameter estimation from data, and when comparing estimated values across different sample groups or experimental designs. In particular, values of only the influential parameters (in fitting the model to the data) should be interpreted and compared.

Local sensitivities are widely used to understand the impact of small perturbations in parameters on the model response, relative to a set of nominal values [17]. Typically, sensitivity derivatives are evaluated one-at-a-time at nominal parameter values [18]. Profile likelihood methods employ a Bayesian framework to evaluate parameter influence, pooling together information after solving multiple optimization problems corresponding to systematically fixing parameters one-at-a-time [19–24]. Recent papers that employ profile likelihood approaches have considered only a small number of parameters and acknowledged potential challenges for models with a moderate number of parameters [19,20,22,23]. Moreover, Markov chain Monte Carlo (MCMC) implementations of Bayesian approaches become costly with an increasing number of model parameters; identifiability analysis is typically carried out first, to reduce the total number of sampled parameters [17,18].

By contrast to the approaches outlined above, local sensitivity-based identifiability analysis uses information from the sensitivity matrix to determine which parameters may be uniquely estimated using the data [17,25] The associated linear algebraic decomposition of the sensitivity matrix yields more detailed information on directional dependencies in the parameter landscape, for both influential and non-influential parameters. Model sloppiness approaches, both Bayesian and standard, also employ such a decomposition, applied to the matrix of sensitivity derivatives of the residual vector (relative to the data) with respect to each model parameter [26–29]. Sloppiness approaches have focused on identifying structural parameter dependencies, the design of experiments and uncertainty quantification [26]. Global structural identifiability analysis searches for algebraic parameter dependencies throughout the entire time domain of the model; several software packages are available for structural identifiability analysis [30,31]. Yet, as noted in our prior study for a physiologically based pharmacokinetic model [17], structural identifiability approaches do not scale well to models with a moderate number of parameters.

In prior work from our group [25], we developed a cooperative enzyme kinetics model for thrombin-mediated fibrin polymerization and a tailored parameter subset selection technique, applying both in the context of one absorbance dataset for fibrin matrix density. These techniques were developed for a single experimental design and identified a (non-influential) subset of unidentifiable parameters containing candidates for model reduction. The subset selection was carried out via sequential iterations of parameter estimation and local sensitivity-based identifiability analysis. Within each iteration, (i) parameters were deemed to be unidentifiable, based on an eigendecomposition involving the local sensitivity matrix, and (ii) the least identifiable parameter was selected as the one in the unidentifiable subset with the lowest residual (in fitting to the data). This (least identifiable) parameter was then fixed at its last estimated value, thus rendering the parameter space to have one less dimension at the next iteration. Iterations were terminated when the smallest eigenvalue exceeded a specified tolerance. Results were compiled in a table summarizing the identifiable parameter subset and cost function value at each iteration of the procedure.

In this study, we use this procedure (and the associated tables) as a foundational component of a data-driven workflow, yielding a more comprehensive and systematic approach to model reduction and subsequent estimation of influential parameters across different designs. When only a single pass (one table) is used, results can be sensitive to initialization of parameter values prior to the first iteration, especially when nominal parameter values are not available. This can impact the obtained subset of unidentifiable parameters, the quality of the curve-fits to the data and the values estimated for the identifiable parameters.

In this study, we introduce randomization of initial parameter values over many realizations into the overall procedure to more comprehensively explore parameter candidates for model reduction, estimate influential parameters using the reduced model, and compare these estimated values across different designs. Local sensitivity-based identifiability analysis is well suited to our overall procedure, given the many iterations and distinct sets of nominal values, about which sensitivities are computed. Our reduced model is determined using one dataset of absorbance measurements for fibrin density. It is then applied to estimate and compare influential parameter values to estimated values for two additional designs in which the system is supplemented with sialic acid at two different concentrations. Through this overall approach, we aim to demonstrate that the reduced model is compatible across experimental designs and that the set of influential parameters, and their estimated values, can be compared without compromising accuracy.

Sialic acid is a negatively charged glycan residue that resides on the surface of most cells and proteins. Previously, we have shown that removal of sialic acid alters clot polymerization kinetics [32]. Clinically, a reduction in sialic acid was linked to increased clot polymerization rates in patients who underwent trauma [33].

We first summarize the data for the three designs used in this study, followed by a brief review of the biochemical reaction system model. We then describe our randomized identifiablility analysis approach, delineating the steps for parameter subset selection involving randomized parameter initialization, computation of local sensitivities and our specific subset selection algorithm. The overall procedure for generating candidate reduced models is then outlined followed by a summary of the statistical test used to compare estimated values of influential parameters. The results of applying these techniques to the control (unmodified) data are then presented. A specific reduced model is systematically identified. This model is then used to estimate and compare the values of parameters deemed to be influential across the three designs.

## 2. Methods

### 2.1. Fibrin matrix polymerization experiments

We first describe the experiments used to evaluate effects of sialic acid on fibrin matrix polymerization. Unmodified adult fibrinogen was purchased from Enzyme Research Laboratories and stored at $-80^{\circ }$C until use. Sialic acid was added to fibrinogen using sialyltransferase [34,35]. To make adult fibrinogen with low levels of sialic acid, 10 mg ml^-1^ commercially available adult fibrinogen (Enzyme Research Laboratories) was mixed with 5 nmol cytidine-$5^{\prime }$-monophospho-N-acetylneuraminic acid (Sigma-Aldrich) and 10 mU ml^-1^α-2,3 sialyltransferase (Sigma-Aldrich) and incubated for 24 hours at 35${}^{\circ }$C. To make adult fibrinogen with medium levels of sialic acid, an additional 5 nmol of cytidine-$5^{\prime }$-monophospho-N-acetylneuraminic acid was added after the previous 24-hour incubation. These samples were then incubated for an additional 24 hours at 35${}^{\circ }$C. After all incubations, solutions were transferred to 300 K Nanosep Centrifugal devices (Pall) and excess sialic acid was spun out for 5 minutes at 10000×g. All samples were stored at $-80^{\circ }$C until sialic acid quantification. To measure sialic acid concentration on modified and unmodified fibrinogen samples, 5 mg ml^-1^ fibrinogen was mixed with 0.025 U ml^-1^ neuraminidase (Sigma-Aldrich) and incubated for 8 hours at 35${}^{\circ }$C. Following incubation, sialic acid was quantified using a sialic acid (NANA) assay kit (Abcam). Unmodified adult fibrinogen had an average sialic acid concentration of 3.6 ± 1.1 nmol while adult fibrinogen + low sialic acid and adult fibrinogen + medium sialic acid had average sialic acid concentrations of 3.7 ± 0.89 nmol and 4.3 ± 0.56 nmol, respectively. To measure fibrin clot polymerization, a well-established absorbance-based assay was used where increases in clot absorbance represent clot formation [36,37]. 2.5 mg ml^-1^ of unmodified adult, adult + low sialic acid and adult + medium sialic acid fibrinogen were mixed with 0.5 U ml^-1^ human alpha thrombin (Enzyme Research Laboratories) in a 96-well plate and clot absorbance (350 nm) was measured over 2 hours via a plate reader. Absorbance values were adjusted by subtracting the initial value (t=0 s); analysis using the model was carried for values starting with the second time point. Figure 1 displays the resulting absorbance curves used as the datasets to develop and apply our model.

**Figure 1.**
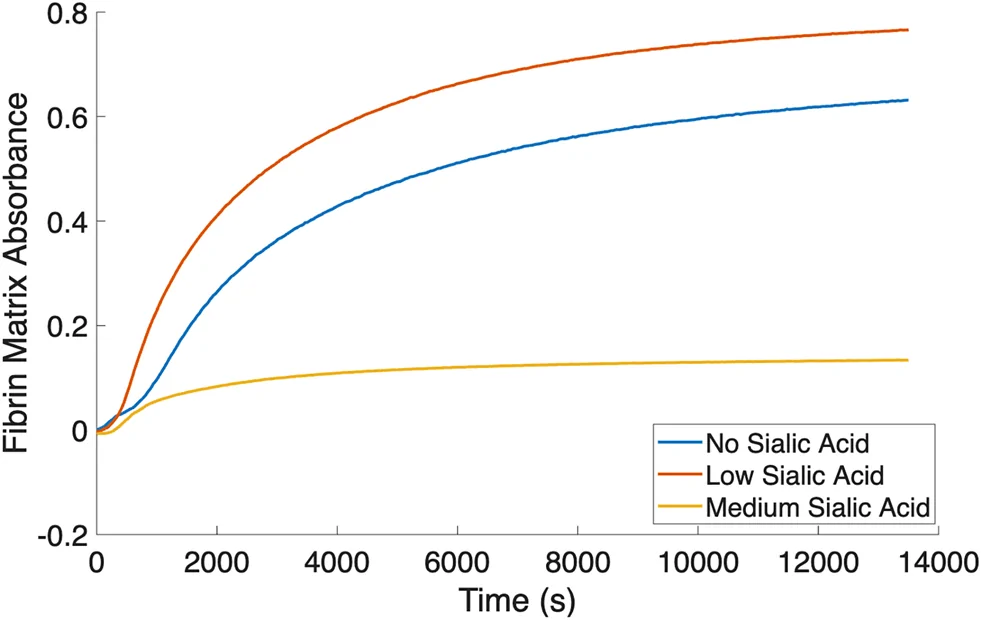
Data from fibrin polymerization experiment for control (no sialic acid) case, low sialic acid and medium sialic acid.

### 2.2. Reaction system and mathematical model

We represent the fibrin matrix polymerization system as the set of biochemical reactions originally presented in [25]. In this system, the transformation of the unbound (dissolved) species fibrinogen into (bound) fibrin matrix is mediated by thrombin through a combination of Michaelis–Menten-type and cooperative enzyme kinetics [25,38]. The first stage of the reaction system (2.1) displays the activation of inactive fibrinogen (Fbn${}_{i}$) into active fibrinogen (Fbn${}_{a}$), mediated by the enzyme thrombin (Thb) (also unbound) and forming the intermediate complex *C*${}_{0}$. Active fibrinogen continues to interact with thrombin and intermediate complexes (*C*${}_{1}$, *C*${}_{2}$) to form the solid-like fibrin matrix (FM) ((2.2) and (2.3)).

(2.1)Fbni+Thb↽−−⇀k−k+⁡C0→k⁡Fbna+Thb,(2.2)Fbna+Thb↽−−⇀k1−k1+⁡C1↽−−⇀k2−k2+⁡Thb+FM,(2.3)Fbna+C1↽−−⇀k3−k3+⁡C2↽−−⇀k4−k4+⁡C1+FM.

Once fibrinogen is activated, the right-most reaction in equation (2.1) occurs rapidly in the forward direction. In addition, active fibrinogen is a dissolved intermediate product that, in turn, reacts to form fibrin matrix. For these reasons, the reverse reaction in the right-most reaction subsystem of equation (2.1) is excluded. The choice of cooperative enzyme kinetics with a few intermediate complexes was based on well-known results that fibrin assembly and matrix accumulation mediated by thrombin exhibit a sigmoidal reaction velocity [7,39]. Our choice corresponds to the simplest case of a Hill-type model [40] with positive cooperativity (Hill coefficient n=2). Lastly, we note that that the FM species in our system is a lumped representation of both fibrin monomers and fibrin matrix. This assumption is tied to the inability of our absorbance-based data measurements to delineate monomers from matrix. Overall, the ability of our model to fit absorbance-based data measuring product (fibrin) accumulation across our designs will determine the validity of these assumptions.

The law of mass action, which is based on first-order reaction kinetics, is used to represent the reactions in equations (2.1)–(2.3) as a system of seven ordinary differential equations (ODEs). From these ODEs, two conservation laws can be identified, as in [25], thus reducing the system to comprise five ODEs and two algebraic relations. All dependent variables in the model represent the concentration of their corresponding species, except for FM which is measured in absorbance units. The resulting system of equations is

(2.4)dFbnidt=−k+Fbni⋅Thb+k−C0,(2.5)dFbnadt=−k1+Fbna⋅Thb+k1−C1−k3+Fbna⋅C1+k3−C2+kC0,(2.6)dThbdt=−k1+Fbna⋅Thb+k1−C1+k2+C1+k−C0−k2−Thb⋅FM−k+Fbni⋅Thb+kC0,(2.7)dC0dt=k+Fbni⋅Thb−k−C0−kC0,(2.8)dFMdt=k2+C1−k2−Thb⋅FM−k4−C1⋅FM+k4+C2,(2.9)C1=−Fbni0+2Thb0−FM0+Fbna+FM−2 Thb+Fbni−C0(2.10)andC2=Fbni0−Thb0+FM0−Fbna−FM+Thb−Fbni.

We have the following initial conditions for the system:

$$\begin{array}{rl} & {\text{Fbn}}_{i}(0)={\text{Fbn}}_{i0},\;{\text{Fbn}}_{a}(0)=0,\;\text{Thb}(0)={\text{Thb}}_{0},\\ & \text{FM}(0)={\text{FM}}_{0},\;C_{j}(0)=0,\;j=0,1,2, \end{array}$$

where ${\text{Fbn}}_{i0}$ is the prescribed initial concentration of fibrinogen, which is all assumed to be inactive. ${\text{Thb}}_{0}$ is the initial concentration of thrombin, which is dependent on the batch obtained for the experiment. Values for both of these initial conditions are specified in §2.1. ${\text{FM}}_{0}$ captures an initial amount of fibrin matrix present a short while after fibrin matrix polymerization initiates; this value is obtained from the experimental data. In addition to these three (non-zero) initial values, equations (2.4)–(2.10) contain n=11 parameters, all of which are (constant) reaction rates. Since the system of ODEs contains a diverse set of characteristic time scales, a stiff ODE solver is used to solve it, numerically.

### 2.3. Parameter estimation

We develop techniques that rely on repeated estimation of model parameters using the given dataset. Moreover, at various stages in our approach, we estimate m<n parameters $p\in R^{m}$ after designating the remaining (n−m) parameters $q\in R^{n-m}$ as being unidentifiable. In all cases, the parameters in p are estimated by minimization of the cost function J(p;q):

(2.11)p∗=arg⁡minp∈R≥0mJ(p;q),(2.12) whereJ(p;q)=(∑i=1N(y(ti,p;q)−yi)2)1/2.

In equation (2.12), $y(t_{i},p;q)$ represents the model response at time $t_{i}$ and $y_{i}$ is the $i^{}$th data point. Our notation delineates parameters that are estimated (p) from those that are fixed at nominal values (q); initially q corresponds to the null set.

The optimization problem is solved with a Nelder–Mead simplex (direct search) algorithm applied to the cost function, as in [25]. This method will be applied for each parameter optimization throughout this paper.

### 2.4. Randomized identifiability analysis

We now describe the use of randomization, parameter optimization and local sensitivity-based identifiability analysis to determine parameters that are influential or non-influential in predicting the model response. Since we are provided with data for only one response variable, it is highly unlikely that all parameters can be uniquely estimated from the data. We refer to parameters that can be reliably estimated as identifiable. Parameters deemed to be unidentifiable are fixed at a nominal value. In our approach, this value is determined from previous iterations of our parameter estimation step (2.11).

Steps A through E in figure 2 depict our overall identifiability analysis process. We begin with order of magnitude estimates for parameters (A) and obtain identifiable parameters, p, and unidentifiable parameters, q, for each randomized realization of the model (B–E).

**Figure 2.**
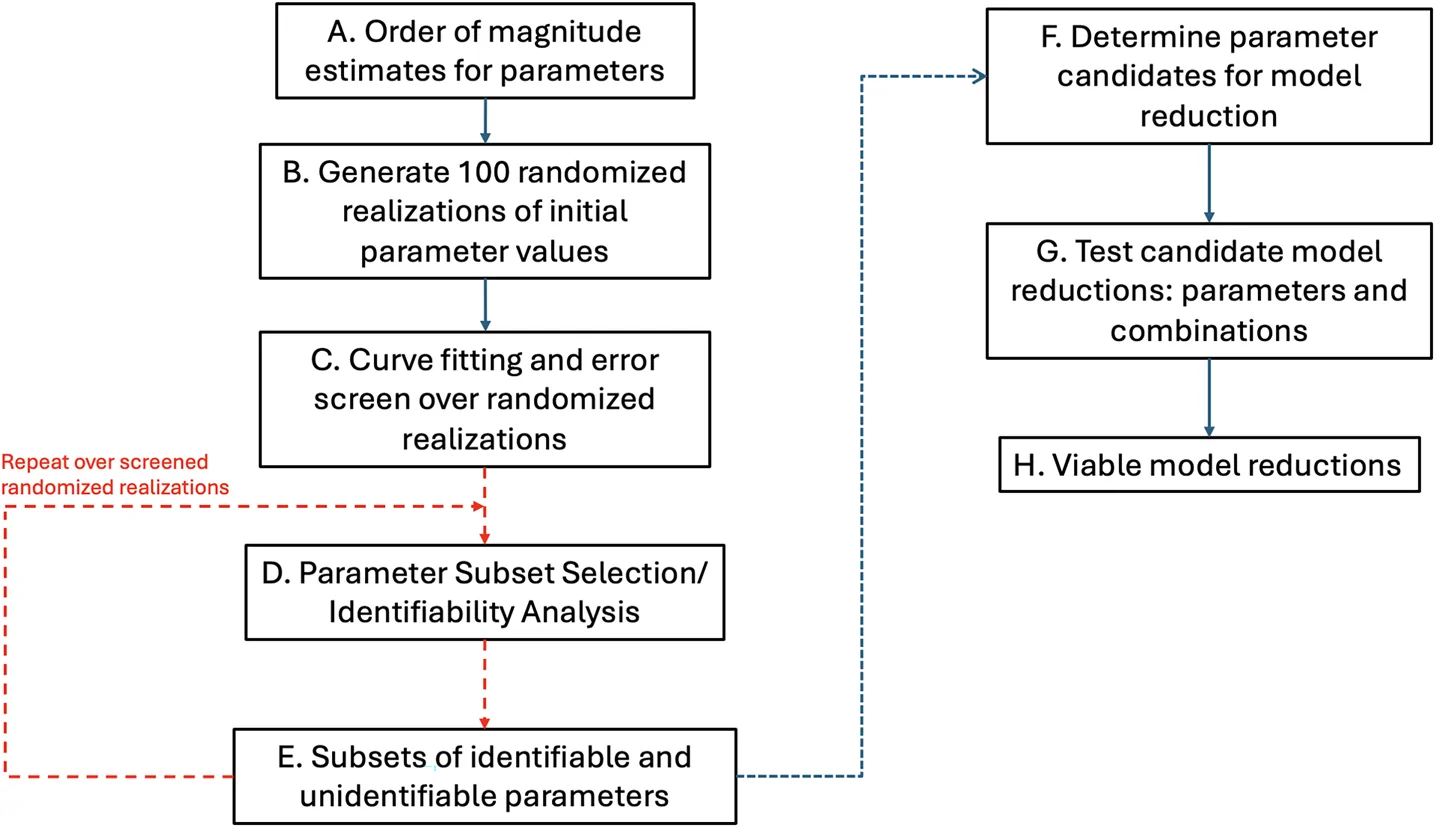
Outline of the process for model reduction based on randomization, identifiability analysis and parameter subset selection. Algorithm 1 represents one iteration of the loop depicted in red.

#### 2.4.1. Randomized parameter initialization

As in [25], we calibrate initial parameter values as powers of 10, based on relative order of magnitude estimates for the reactions rates (table 1). For the dataset (§2.1), we used the following initial values to comprise $p_{0}$.

**Table 1.** Initial power of 10 values ($p_{j}^{0}$) for parameters.

parameter	initial value	units
k	1000	${\text{s}}^{-1}$
$k^{-}$	100	${\text{s}}^{-1}$
$k_{1}^{-}$	100	${\text{s}}^{-1}$
$k_{2}^{-}$	0.1	${\text{(Ms)}}^{-1}$
$k_{3}^{-}$	10	${\text{s}}^{-1}$
$k_{4}^{-}$	100	${\text{(Ms)}}^{-1}$
$k^{+}$	0.01	${\text{(Ms)}}^{-1}$
$k_{1}^{+}$	0.1	${\text{(Ms)}}^{-1}$
$k_{2}^{+}$	100	${\text{s}}^{-1}$
$k_{3}^{+}$	1	${\text{(Ms)}}^{-1}$
$k_{4}^{+}$	10	${\text{s}}^{-1}$

For each initial parameter $p_{j}^{0}$ in $p_{0}$, we generate

(2.13) $$\begin{array}{r} p_{j}=p_{j}^{0}\cdot 10^{-1+2\alpha_{j}}\;(j=1,\ldots ,n), \end{array}$$

where each $\alpha_{j}$ is a randomly sampled from U(0,1). This process is independently repeated to generate r=100 realizations of initial parameter values (step B). In step C, we perform an initial curve fit, independently, on each set of parameters via equation (2.11) to generate the realizations of the model. We then conduct an error-screen over the realizations to eliminate fits of lower accuracy, before implementing parameter subset selection on the remaining cases.

#### 2.4.2. Local sensitivities

Our identifiablity analysis is based on the sensitivity matrix (S) whose entries are the local sensitivities at a specified set of N times ($t_{i}$):

(2.14) $$\begin{array}{r} S_{ij}=\frac{\partial f}{\partial p_{j}}(t_{i},p)\;(i=1,2,\ldots ,N,\;j=1,2,\ldots ,n). \end{array}$$

These derivatives are evaluated numerically using the complex step approximation of the scaled local sensitivities:

(2.15) $$\begin{array}{r} \frac{\partial f}{\partial p_{j}}=2\alpha p_{j}^{*}\cdot \frac{\text{imag}(f(t,p;p_{j}+h\hat{i}))}{h}\;({\hat{i}}^{2}=-1), \end{array}$$

where $p_{j}^{*}$ represents the parameter value. α=0.1, is a scaling parameter [25] and h is a specified step size. The derivative approximation (2.15) is robust to subtractive cancellation and exhibits better numerical convergence compared to standard finite difference approximations [18].

The sensitivity matrix is the cornerstone of our identifiability analysis, as its associated linear algebraic decompositions reveal information about influential and non-influential directions in the parameter landscape.

#### 2.4.3. Parameter subset selection

We combine parameter estimation techniques and linear algebraic properties of S to develop an algorithm for determining subsets of unidentifiable parameters, among the full set of model parameters, for each realization (algorithm 1). This corresponds to steps D and E in figure 2 and is based on the singular value decomposition (SVD) of S and its associated singular values ($\sigma_{j},\;j=1,\ldots ,m$).

**Algorithm 1 Parameter subset selection.**

**Figure:**
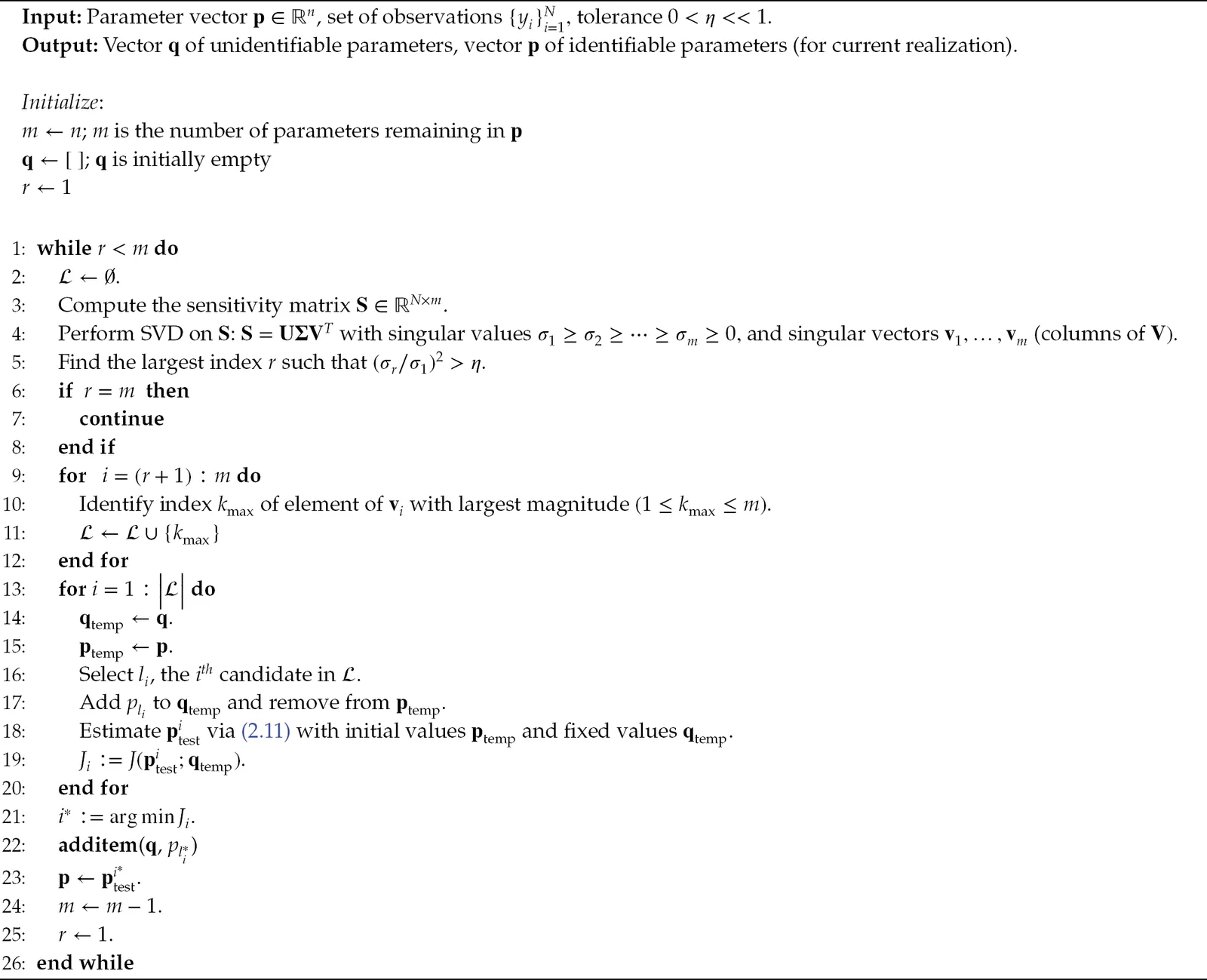

For each random realization, we begin with the corresponding parameter vector $p\in R^{n}$ and q=[ ]; p is most commonly the first estimate for the full set of model parameters obtained using equation (2.11). Since the size of p changes as the algorithm proceeds, we will refer to its size as m from now on; initially, m=n.

In algorithm 1, at the beginning of each iteration (lines 2–25), we reset the list L that will hold candidate unidentifiable parameters (line 2). We evaluate the sensitivity matrix $S\in R^{N\times m}$ (2.14)–(2.15) around the current parameter values (p) and compute its SVD (line 4). We normalize the singular values with respect to their largest value, locating the index (r) of the smallest singular value above a specified tolerance η (line 5). When all of the squared singular values of S are greater than η, r=m, and all parameters in p are identifiable and the algorithm terminates (lines 6–8).

For each normalized singular value less than η, the algorithm examines the corresponding singular vector to find the index ($k_{\text{max}}$) of the element with the largest magnitude. The index $k_{\text{max}}$ is added to L (lines 9–12). Each parameter corresponding to the candidate indices in L is then independently fixed at its current value in p, and parameter estimation is run on the remaining parameters. This procedure is repeated for each candidate parameter (lines 13–20). We choose the parameter candidate yielding the lowest cost when fixed and designate this parameter to be unidentifiable. This parameter is then removed from p and added to q (lines 22–23). These vectors p and q are then used in the next iteration of the while loop.

Once algorithm 1 concludes, we have a parameter vector of k unidentifiable parameters for the current realization, $q\in R^{k}$ and a corresponding vector of n−k identifiable parameters $p\in R^{n-k}$.

We apply algorithm 1 for every random realization, as represented by the red loop (steps D and E) in figure 2. We then conduct another error-screen with a lower threshold than in step C. We eliminate realizations with cost above the threshold to ensure that we only interpret parameter identifiability information from high quality fits to the data. At the end of this procedure, we have independent subsets of identifiable and unidentifiable parameters for every screened realization.

### 2.5. Model reduction

We refer to model reduction as the process of setting some unidentifiable parameter values to zero and no longer estimating those parameters. This corresponds to removing the associated reactions from the reaction system (2.1)–(2.3). We now describe how the results from our randomized identifiability analysis are used for model reduction.

When algorithm 1 is applied for parameter subset selection, we obtain a subset of unidentifiable parameters for each random realization. We pool and analyse these unidentifiable parameter subsets to identify candidate parameters for model reduction (step F). For structural reasons, we only consider reverse reaction rates as candidates; the removal of forward reaction rates would impede the forward progress of the reaction system.

We select candidates for model reduction as parameters that are found to be unidentifiable in at least 50% of the realizations. This level was chosen to preserve wide exploration of parameter candidates for removal of specific reactions from our overall system. After choosing candidate parameters, we then test the viability of the corresponding model reductions with one iteration of parameter estimation via equation (2.11). We test each candidate parameter independently as well as all possible combinations of the candidates (step G).

For a specific candidate model reduction, the parameter candidate(s) are removed from the vector p obtained via algorithm 1 for a given realization. The removed parameters are set to zero in the model. The remaining entries in p are then used as initial values in a new solution of equation (2.11). This process is repeated over all of the realizations that met the error-screen in step D. We consider the interquartile range of errors for the associated curve fits to determine the set of viable model reductions (step H).

Once a reduced model is determined, steps A through E (figure 2) are repeated with the aim of identifying influential parameters. Parameters that are consistently not unidentifiable are deemed to be influential and estimated and compared across the three experimental designs.

### 2.6. Statistical testing

When comparing parameter estimate values across experimental designs, we use the two-tailed Mann–Whitney test. The Mann–Whitney test distinguishes whether two samples X and Y come from distributions with the same median. Our samples will be estimated parameter values over all successful realizations. For each parameter, we apply the test pairwise on corresponding samples across different experimental designs. Specifically, the two-tailed test assumes the following null and alternative hypotheses [41]:

(2.16)H0=P(X<Y)=12(2.17)andH1=P(X<Y)≠12.

For a sample X of size $N_{x}$ and a sample Y of size $N_{y}$, we first order all of the observations by magnitude and assign ranks. We then compute the sum of ranks for each sample, $R_{x}$ and $R_{y}$. The Mann–Whitney test statistic, U, can be computed as follows [41]:

(2.18)Ux=NxNy+Nx(Nx+1)2−Rx,(2.19)Uy=NxNy+Ny(Ny+1)2−Ry(2.20)andU=min{Ux,Uy}.

The test statistic U is compared to Mann–Whitney tables, where critical U values can be found for specified *p*-values.

## 3. Results

### 3.1. Implementation

We used our mathematical model (2.4)–(2.10) with n=11 initial parameters in applying the randomization, parameter subset selection and model reduction techniques outlined in §2. All implementations were carried out in MATLAB 2025a. For parameter estimation, we used fminsearch to minimize the cost function (2.12), with function and step size tolerances of $10^{-6}$ and $10^{-5}$, respectively. We used the stiff ODE solver ode15s to numerically solve the system of ODEs in our model. The process detailed in figure 2 was first implemented with our model (2.4)–(2.10) for the control data shown in figure 1.

#### 3.1.1. Randomization

The random initialization was carried out via equation (2.13), yielding 100 realizations (figure 2, steps A and B). For each realization, we ran one iteration of curve-fitting and implemented the initial screening process to remove poor fits to the data (figure 2, step C). We used the screening tolerance

(3.1) $${\left(\sum_{i=1}^{N}(y(t_{i},p;q)-y_{i}{)}^{2}\right)}^{1/2}<\epsilon$$

with ϵ=2. Based on the number of data points, this choice corresponds to a mean squared error of roughly 0.013 relative to the range of the data in figure 1. It is also intended to screen out cases where the model fails to replicate the general behaviour of the data; the tolerance will be further reduced at later stage in our overall procedure. After initial screening, the control dataset yielded 58 (out of 100) successful realizations.

#### 3.1.2. Parameter subset selection

The successful realizations were used for the identifiability analysis and parameter subset selection (figure 2, steps D and E). In executing algorithm 1 (step D), we prescribed a tolerance $\eta =10^{-8}$. Sample tables illustrating the identifiability analysis procedure for two realizations are shown in table 2. In table 2a, the unidentifiable parameter vector q contained the six parameters $\left\{k,k^{-},k_{1}^{-},k_{3}^{-},k_{4}^{-},k_{3}^{+}\right\}$ with a final error J=0.0423. In the other case shown (table 2b), the unidentifiable parameter vector q contained the five parameters $\left\{k,k^{-},k_{1}^{-},k_{3}^{-},k_{2}^{+}\right\}$ with a final error J=0.0736. The corresponding fits to the data using the final parameter values in each column are shown in figure 3.

**Figure 3.**
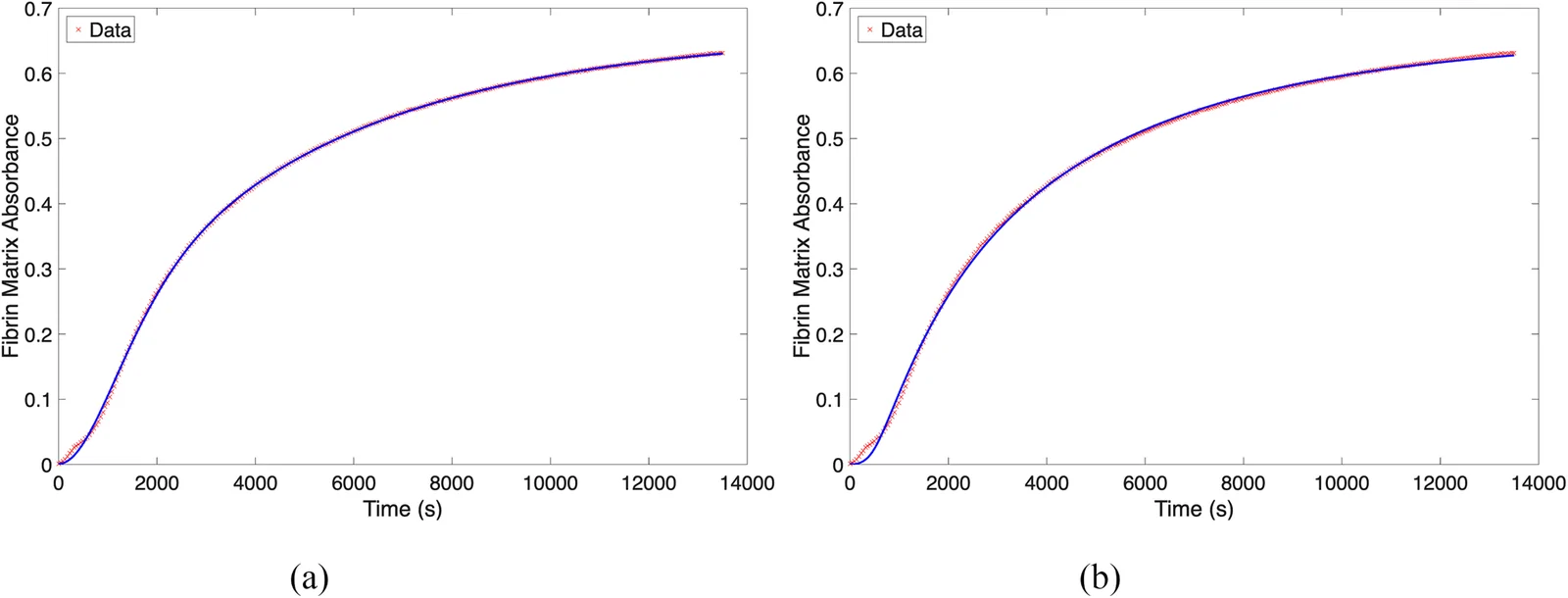
Corresponding model fits for the two cases shown in table 2 with the control dataset. Fibrin matrix absorbance for parameter values in the final row of: (a) table 2a and (b) table 2b.

**Table 2.** Results of identifiability analysis for two sample realizations ((a) and (b)) in the application of algorithm 1. A square in a parameter’s column indicates that it has been deemed unidentifiable. In subsequent iterations, its value is fixed at the estimate from the previous iteration.

iter.	k	$k^{-}$	$k_{1}^{-}$	$k_{2}^{-}$	$k_{3}^{-}$	$k_{4}^{-}$	$k^{+}$	$k_{1}^{+}$	$k_{2}^{+}$	$k_{3}^{+}$	$k_{4}^{+}$	error
—	1000	100	100	0.1	10	100	0.01	0.1	100	1	10	—
(a)												
**0**	1428	404.2	520.5	1.330E-3	1.811	109.8	3.400E-4	0.09658	15.36	2.528	6.554	0.0828
**1**	1947	99.97	708.6	1.362E-3	1.778	114.4	3.610E-4	0.0951	14.29	3.426	6.935	0.0729
**2**	◻	1073	311.4	1.158E-7	1.636	125.5	6.247E-4	0.07025	4.682	5.972	8.230	0.0423
**3**	◻	1078	313.1	1.157E-7	1.635	125.6	6.261E-4	0.07051	4.679	◻	8.237	0.0423
**4**	◻	◻	313.2	1.162E-7	1.635	125.6	6.262E-4	0.07053	4.678	◻	8.238	0.0423
**5**	◻	◻	315.7	◻	1.637	125.7	6.263E-4	0.07114	4.673	◻	8.248	0.0423
**6**	◻	◻	315.0	◻	◻	125.7	6.264E-4	0.07096	4.672	◻	8.249	0.0423
**7**	◻	◻	◻	◻	◻	125.7	6.264E-4	0.07096	4.672	◻	8.249	0.0423
**8**	◻	◻	◻	◻	◻	◻	6.266E-4	0.07102	4.666	◻	8.252	0.0423
(b)												
**0**	725.6	46.96	138.0	0.9291	2.185	347.6	5.100E-4	0.1519	335.8	1.195	35.72	0.2556
**1**	620.9	62.28	134.8	1.053	1.733	0.05646	5.885E-4	0.1491	312.3	3.561	9.870	0.2415
**2**	1554	◻	65.52	1.897	2.880	0.05685	6.835E-4	0.1184	228.4	35.27	1.466	0.2190
**3**	◻	◻	72.81	2.446	0.1364	0.1614	8.218E-4	0.1681	147.8	24.26	1.588	0.2018
**4**	◻	◻	◻	10.56	1.073	10.266	1.761E-3	0.5947	7.289E-4	82.08	2.874	0.1085
**5**	◻	◻	◻	◻	0.7601	32.52	1.978E-3	0.08765	1.786E-3	99.05	2.238	0.0741
**6**	◻	◻	◻	◻	◻	35.92	1.933E-3	0.09060	1.673E-3	97.61	2.362	0.0736
**7**	◻	◻	◻	◻	◻	35.92	1.932E-3	0.09062	◻	97.61	2.362	0.0736

The error J across the 58 realizations considered exhibited significant variation (figure 4a). By reducing the tolerance in equation (3.1) by a factor of 20, we retained 24 (of 58 cases), enabling the interpretation of parameter identifiability using cases with fits of greater accuracy (figure 4b). Absolute average residual curves are also shown in figure 4c.

**Figure 4.**
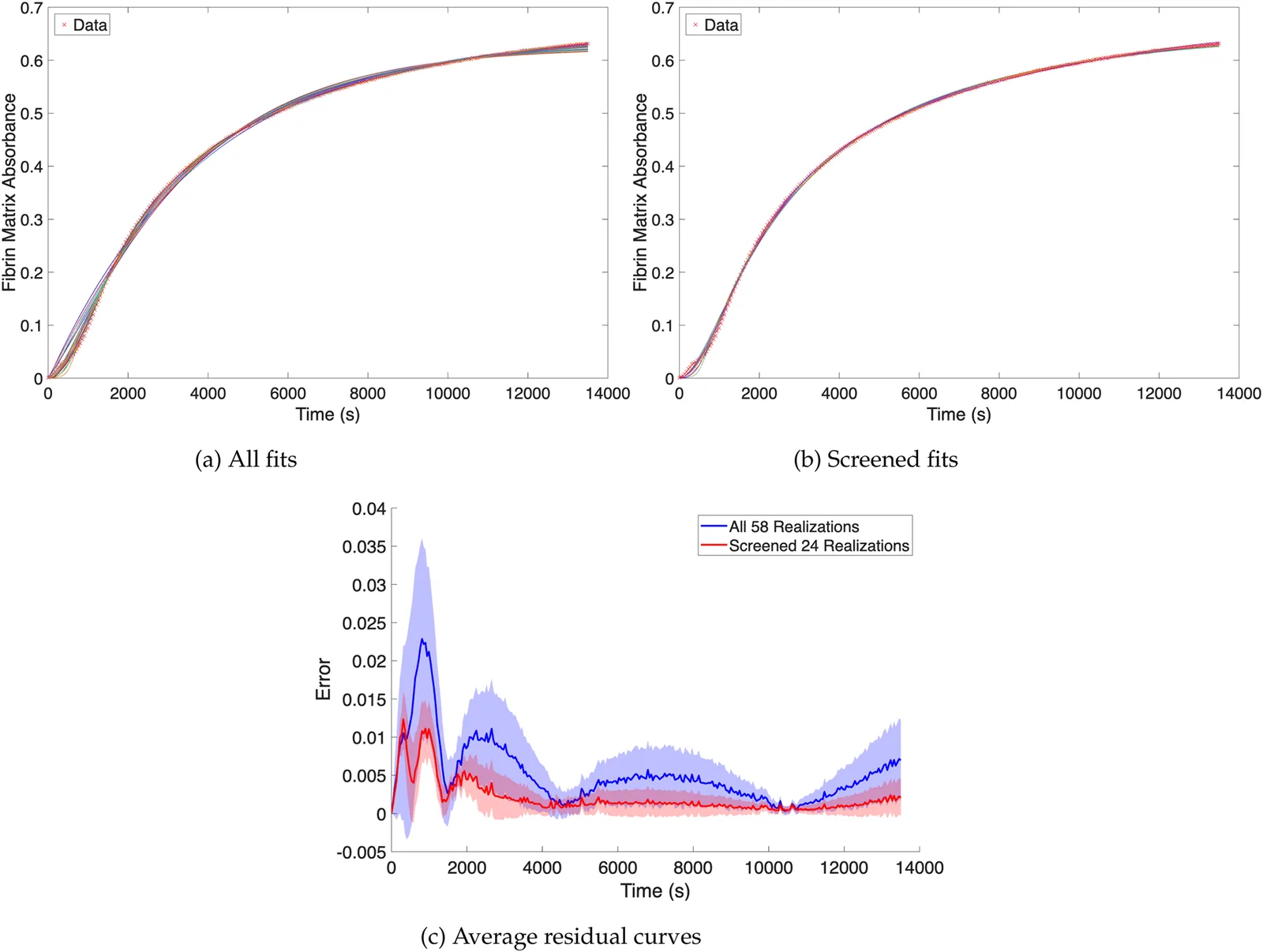
Curve fits of fibrin matrix absorbance to control data: (a) across all 58 realizations with ϵ=2, (b) across 24 realizations after further screening by reducing ϵ by a factor of 20, and (c) average absolute residual curves versus time (shading denotes one standard deviation above and below the mean).

To consolidate the information gained from our identifiability analysis, we determined the unidentifiable instances of each model parameter across these 24 cases (table 3). We observe that parameters k, $k_{3}^{-}$, $k^{-}$, $k_{3}^{+}$, $k_{4}^{+}$ and $k_{1}^{-}$ all had unidentifiable instance percentages above 70%; the next highest percentage is 54.2%. Among these six parameters, we note that $k_{3}^{-}$, $k^{-}$ and $k_{1}^{-}$ are reverse reaction rates, with corresponding percentages of 95.8%, 87.5% and 70.8%.

**Table 3.** Instances and percentages of unidentifiability for each parameter over the 24 realizations that passed the second error screen.

parameter	unidentifiable instances	%
k	23	95.8
$k^{-}$	21	87.5
$k_{1}^{-}$	17	70.8
$k_{2}^{-}$	7	29.2
$k_{3}^{-}$	23	95.8
$k_{4}^{-}$	13	54.2
$k^{+}$	4	16.7
$k_{1}^{+}$	1	4.2
$k_{2}^{+}$	12	50.0
$k_{3}^{+}$	21	87.5
$k_{4}^{+}$	21	87.5

### 3.2. Model reduction

The presence of consistently unidentifiable reaction rates suggests the potential for model reduction. We first consider the forward reaction rate parameters $k_{3}^{+}$ and $k_{4}^{+}$. To preserve forward progress in our reaction system, elimination of these two parameters requires removal of the reaction subsystem (2.3). When our testing procedure was attempted with a model reduced in this manner, errors increased, with a median of 1.96 and interquartile range (1.82–2.04). Among the reverse reaction rates, all model reductions in which $k_{4}^{-}$ was set to zero yielded errors above our threshold. Consequently, we considered only $k^{-},\;k_{1}^{-}\;$ and $k_{3}^{-}$ as our parameter candidates for model reduction. Considering all possible combinations of these parameters yields seven candidate model reductions: setting to zero $\left\{k^{-}\right\}$, $\left\{k_{1}^{-}\right\}$, $\left\{k_{3}^{-}\right\}$, $\{k^{-}$, $k_{1}^{-}\}$, $\{k^{-}$, $k_{3}^{-}\}$, $\{k_{1}^{-}$, $k_{3}^{-}\}$ and $\{k^{-},\;k_{1}^{-}$, $k_{3}^{-}\}$.

We tested each candidate model reduction independently, as described in the last paragraph of §2.5. This yielded 24 curve fits to the data, of which a subset was viable for each of the seven reduced models; fits were deemed not viable when the optimization algorithm failed. Figure 5 shows the corresponding errors (J) over the realizations for each candidate model reduction compared to the control dataset. The corresponding medians and interquartile ranges of J for each candidate model reduction are shown in table 4.

**Figure 5.**
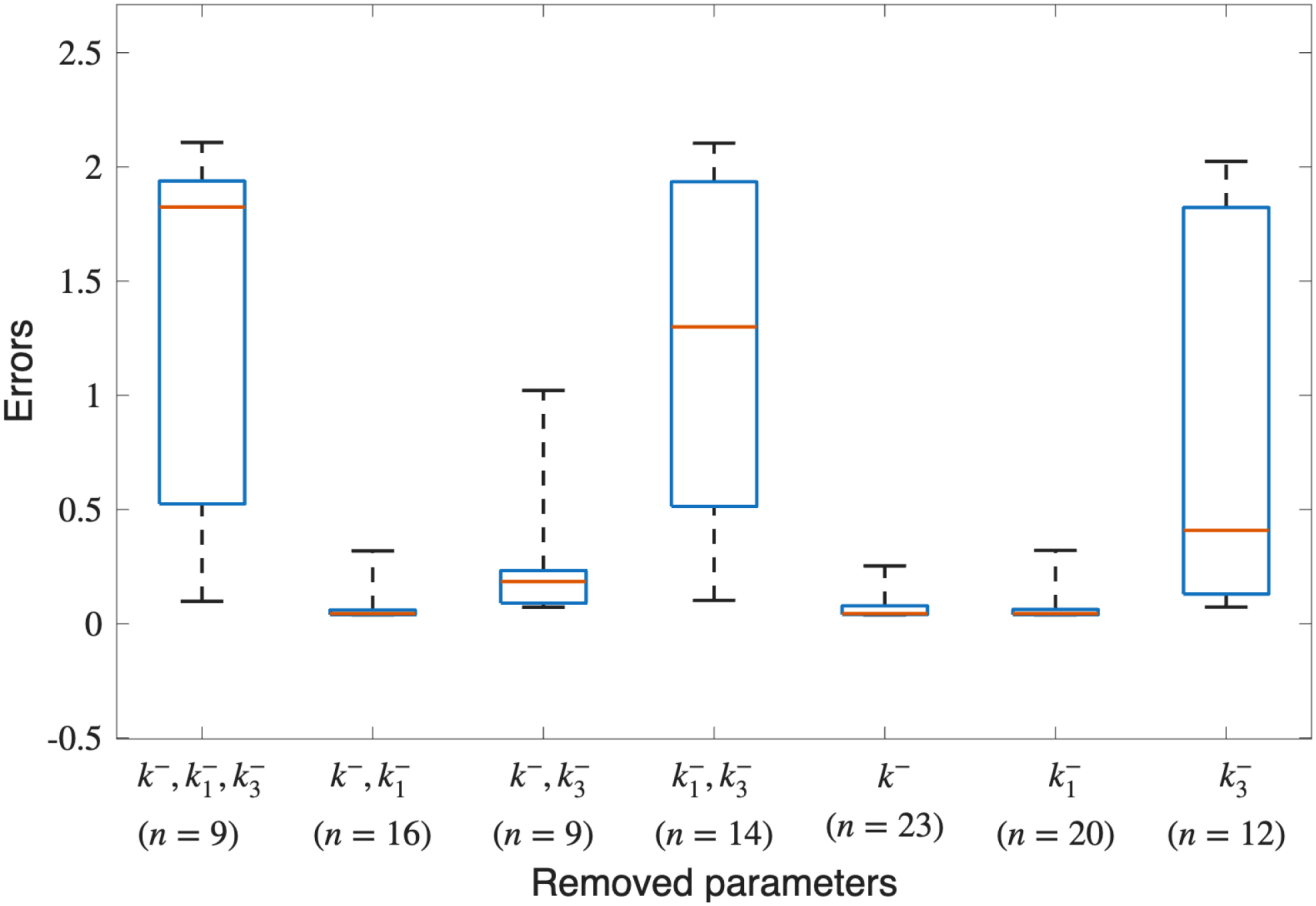
Box and whisker plot of errors associated with each of the seven candidate model reductions, over the realizations for control data.

**Table 4.** Interquartile ranges and medians of the error for each candidate model reduction when tested over realizations for the control data.

parameters removed	median error	interquartile range
$k^{-},\;k_{1}^{-},\;k_{3}^{-}$	1.824	(0.524–1.938)
$k^{-},\;k_{1}^{-}$	0.044	(0.039–0.060)
$k^{-},\;k_{3}^{-}$	0.185	(0.090–0.233)
$k_{1}^{-},\;k_{3}^{-}$	1.299	(0.514–1.935)
$k^{-}$	0.045	(0.041–0.078)
$k_{1}^{-}$	0.044	(0.040–0.062)
$k_{3}^{-}$	0.409	(0.130–1.822)

For the three model reductions where $k^{-}$ is removed, $k_{1}^{-}$ is removed and both $k^{-}$ and $k_{1}^{-}$ are removed, the first three quartiles are below our error tolerance for an acceptable fit (J<ϵ/20). All four model reductions involving $k_{3}^{-}$ had a median error above this tolerance. Thus, we concluded that there were three viable model reductions, i.e. setting to zero $\left\{k^{-}\right\}$, $\{k_{1}^{-}$} and $\{k^{-}$, $k_{1}^{-}\}$, and proceeded with the model reduction eliminating the reverse reactions for $k^{-}$ and $k_{1}^{-}$. This defines our reduced model, which contains nine parameters.

### 3.3. Identifying and estimating influential parameters

We shift our focus to determining influential parameters in the reduced model, in order to estimate their values and compare these values across different designs. With the reduced model, we first repeated the process detailed in steps A through E of figure 2 with the control data. Specifically, we used the nine parameter values from the lowest error realization of the reduced model in §3.2 as our baseline parameter values for randomization across 100 realizations. After both stages of error screening and the identifiability analysis, the secondary error screen (J<ϵ/20) yielded 39 (successful) realizations (figure 6). Table 5 displays the number of times that each parameter was considered not unidentifiable across these 39 cases; parameters that were consistently not unidentifiable based on algorithm 1 were deemed to be influential.

**Figure 6.**
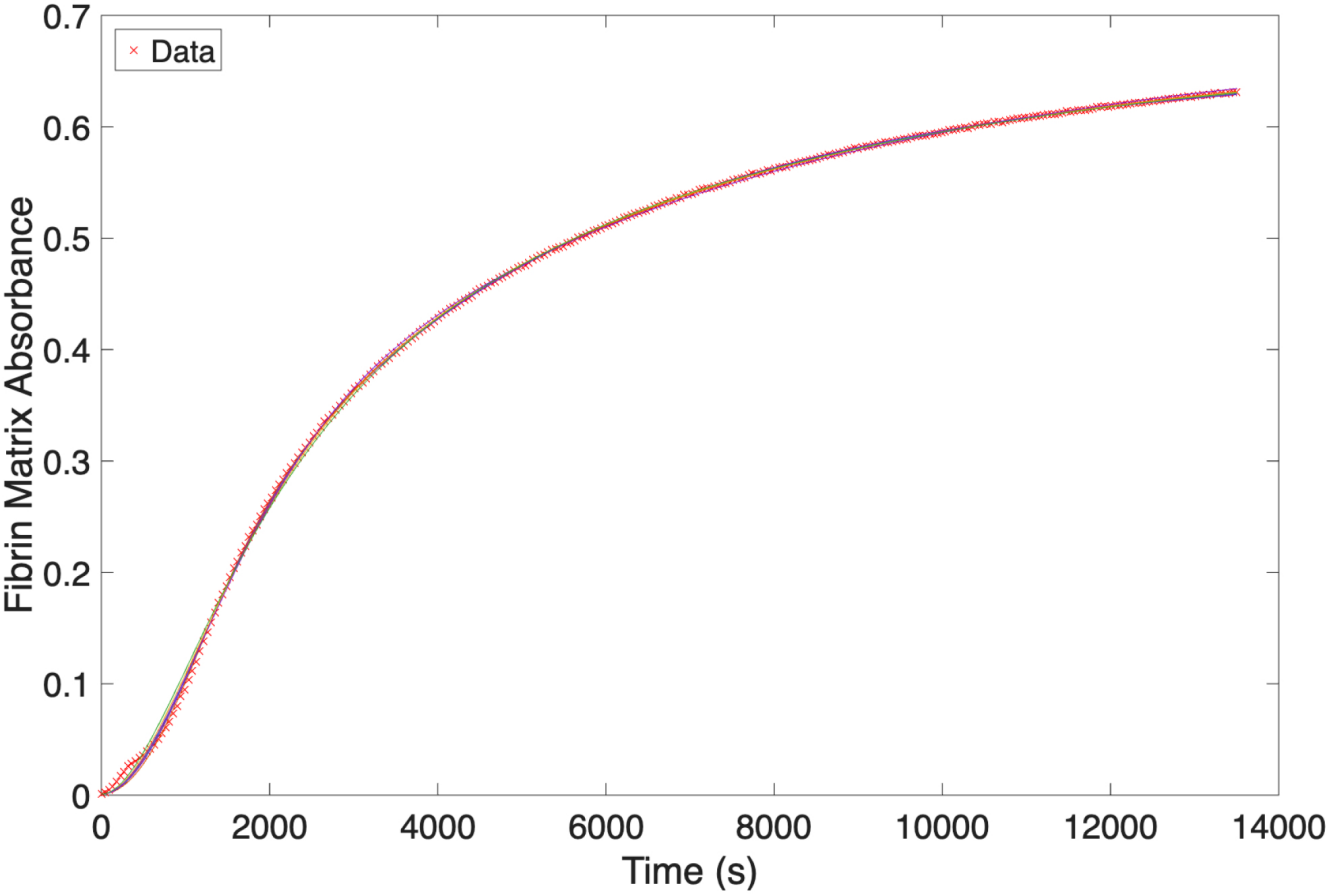
Curve fits of fibrin matrix absorbance to control data using the reduced model across 39 realizations based on an error screen with ϵ/20.

**Table 5.** Instances and percentages where each parameter is deemed to be influential across 39 screened randomized realizations of the reduced model.

parameter	influential instances	%
k	0	0.0
$k_{2}^{-}$	17	43.6
$k_{3}^{-}$	23	59.0
$k_{4}^{-}$	16	41.0
$k^{+}$	39	100.0
$k_{1}^{+}$	39	100.0
$k_{2}^{+}$	21	53.8
$k_{3}^{+}$	3	7.69
$k_{4}^{+}$	1	2.56

We observe that parameters $k^{+}$ and $k_{1}^{+}$ are influential in 100% of the 39 realizations (table 5). The next most consistently influential parameter ($k_{3}^{-}$) occurs in 59% of instances. Owing to this substantial drop, we deem parameters $k^{+}$ and $k_{1}^{+}$ to be the most influential, and thus are our chosen parameters for estimation. We report the following means for the identifiable parameters:

$$\begin{array}{rl} {\bar{k}}^{+} & =3.579\cdot 10^{-4}\;(\text{Ms}{)}^{-1}\\ \text{and}\;{\bar{k}}_{1}^{+} & =1.336\cdot 10^{-3}\;(\text{Ms}{)}^{-1}, \end{array}$$

with corresponding standard deviations $5.12\cdot 10^{-5}$ and $3.95\cdot 10^{-4}$, respectively.

### 3.4. Comparison across experimental designs

Based on the hypothesis that our reduced model is applicable to the other two designs outlined in §2.1, we carried out a parameter estimation procedure for these additional designs. We repeated the procedure outlined in the first paragraph of §3.3 (steps A through E of figure 2), using the orders of magnitude from table table 1 as baseline values; again, 100 realizations of initial parameter values were generated. We implemented the initial error screen (3.1) with ϵ=2 and passed the (successful) realizations through algorithm 1 and implemented the stricter error screen using ϵ/20.

For consistency with the process implemented on the standard control data in §3.3, we then selected the lowest error realization for both datasets. Using each of these two parameter vectors as the baseline, we repeated the randomization to generate 100 realizations of initial parameter values. We then ran algorithm 1 and the error screen again over these realizations for each dataset, yielding 68 successful realizations for the low sialic acid data and 62 successful realizations for the medium sialic acid data. Figure 7 demonstrates that the resulting fits to the data for both alternative designs were excellent.

**Figure 7.**
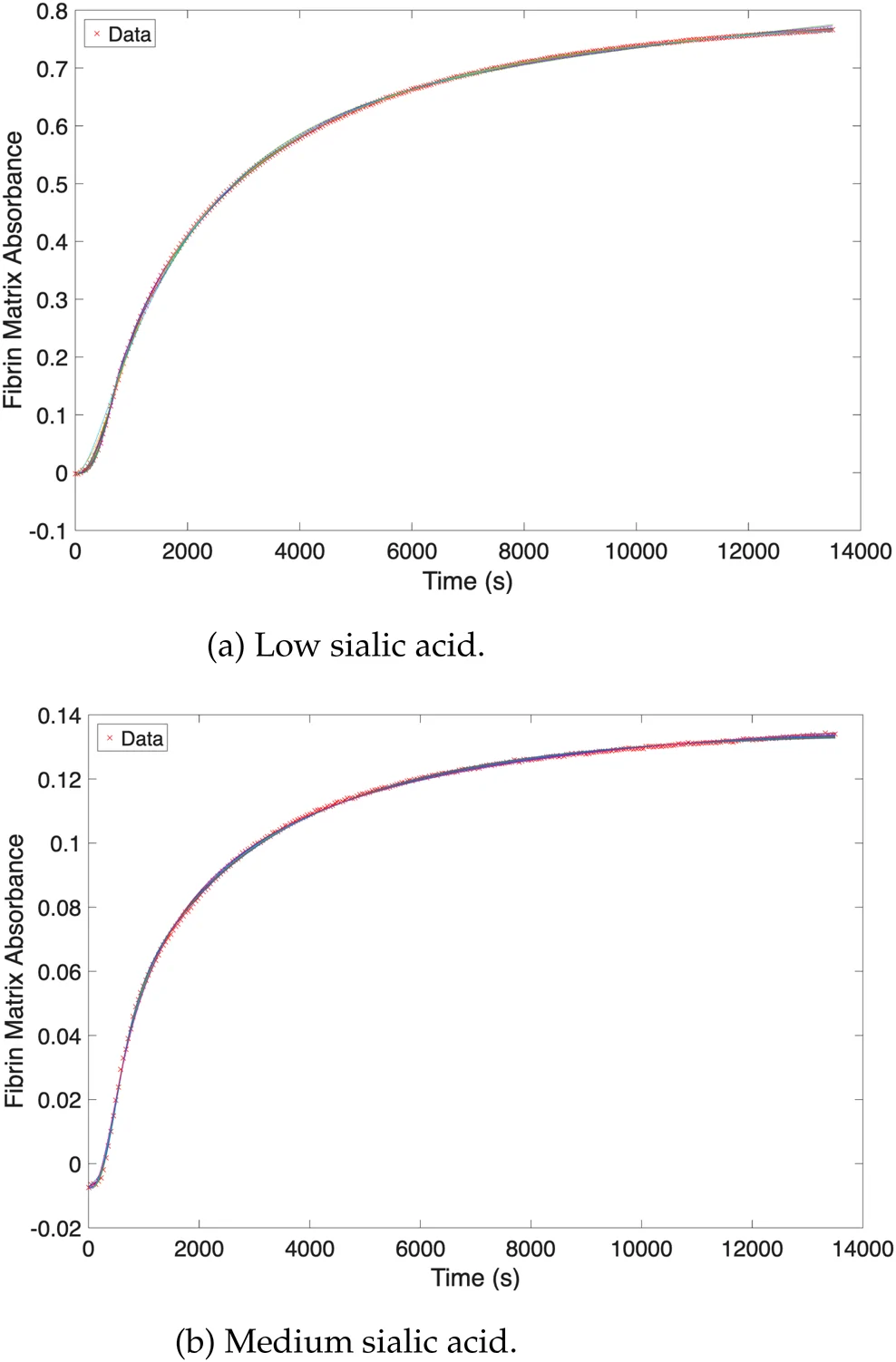
Curve fits of fibrin matrix absorbance using the reduced model based on an error screen with ϵ/20 for: (a) the low sialic acid data (68 realizations) and (b) the medium sialic acid data (62 realizations).

To verify that the two parameters $k^{+}$ and $k_{1}^{+}$ are also influential for these two designs, we determined the percentage of realizations in which each parameter is deemed influential for the three different experimental designs. For convenience, this information is compared for all three cases in table 6.

**Table 6.** Percentage of realizations where each parameter is deemed influential over $\tilde{s}$ realizations for the control data, low sialic acid data and medium sialic acid data.

parameter	control	low	medium
	$\tilde{s}=39$	$\tilde{s}=68$	$\tilde{s}=62$
k	0.0	95.6	100.0
$k_{2}^{-}$	43.6	64.7	100.0
$k_{3}^{-}$	59.0	60.3	3.2
$k_{4}^{-}$	41.0	77.9	67.7
$k^{+}$	100.0	100.0	100.0
$k_{1}^{+}$	100.0	98.5	100.0
$k_{2}^{+}$	53.8	33.8	38.7
$k_{3}^{+}$	7.7	27.9	16.1
$k_{4}^{+}$	2.6	83.8	3.2

We observe a very high occurrence frequency for $k^{+}$ and $k_{1}^{+}$, namely five values of 100% with a sixth value of 98.5%. No other parameter was consistently influential across all three cases. Hence, we focus on $k^{+}$ and $k_{1}^{+}$ as two influential parameters that can be compared across all three designs. Figure 8 displays the middle 50% of parameter estimates for these two parameters; the corresponding interquartile ranges are shown in table 7.

**Figure 8.**
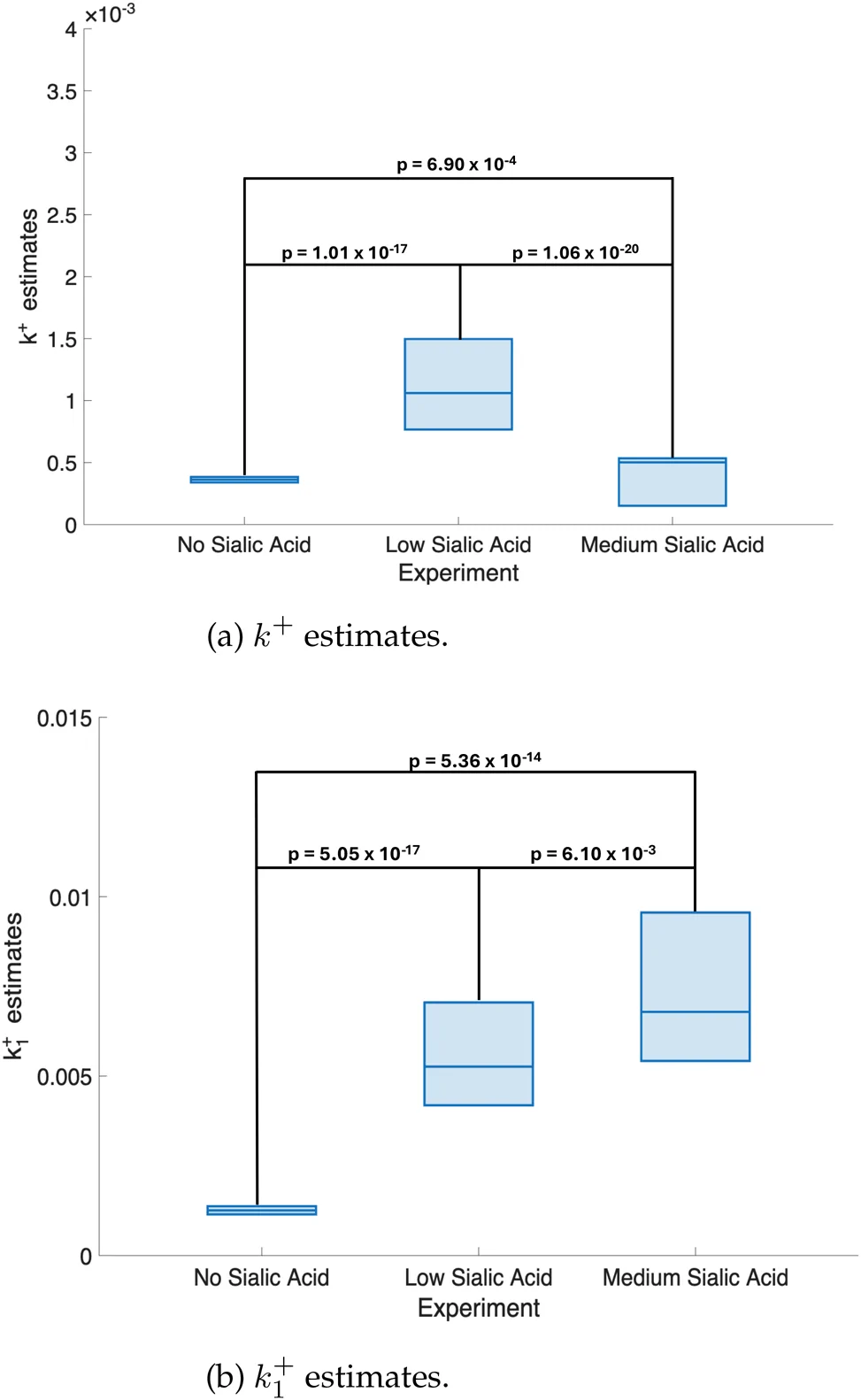
Middle 50% of estimated parameter values for the top two influential parameters, across the three experimental designs: (a) parameter $k^{+}$ and (b) parameter $k_{1}^{+}$.

**Table 7.** Interquartile ranges of parameter estimates across experimental designs for the control data, low sialic acid data and medium sialic acid data.

dataset	$k^{+}\cdot 10^{-4}\;(\text{Ms}{)}^{-1}$	$k_{1}^{+}\cdot 10^{-3}\;(\text{Ms}{)}^{-1}$
control	(3.39–3.84)	(1.15–1.37)
low	(7.67–14.97)	(4.19–7.05)
medium	(1.51–5.34)	(5.42–9.56)

We applied the statistical test outlined in §2.6 to determine significance of parameter estimates across experimental designs. We used MATLAB’s ranksum function with a *p*-value of 0.05. For each influential parameter, we independently applied the test pairwise across the three experimental designs. We observed in all cases that p<0.05; when applied to the middle 50% of the estimates, we found a maximum $p=1.6\cdot 10^{-6}$. When applied to the entire set of realizations, the maximum *p*-value was $p=6.1\cdot 10^{-3}$. The biological significance of these findings is summarized in the next section.

## 4. Discussion

In this study, we have developed techniques based on a randomized local sensitivity-based identifiability analysis approach and integrated them into a tailored workflow for parameter subset selection, model reduction, estimation of influential parameters and comparison across different experiment-based designs. The reduced model determined through this process was applied to analyse fibrin matrix absorbance data across three experimental designs to investigate the impact of sialic acid on fibrin polymerization. Our approach enabled testing for statistical significance between different designs, demonstrating differences for several identifiable (and hence influential) reaction rates across designs with and without sialic acid. Our model contains several simplifying assumptions about fibrin matrix assembly and owing to using absorbance-based measurements. Nevertheless, validity of our model is evidenced by our ability to systematically identify a reduced model using only the control case, and then accurately fit absorbance data and estimate influential reaction rates across all three designs. Moreover, our attempt to reduce the model from three to two intermediate complexes failed to accurately fit our data, thus providing further evidence that the scope and level of detail in our model are well aligned with our experimental data.

Our results provide some insight for how sialic acid concentration on fibrinogen impacts clot polymerization. While previous experiments indicate sialic acid impacts clot polymerization kinetics, the exact mechanism for these differences remains elusive [32]. In developing the clot polymerization model [25], we assigned specific clot polymerization steps to the reaction subsystems in equations (2.1)–(2.3). Regarding equations (2.1) and (2.2), where the parameters $k^{+}$ and $k_{1}^{+}$ were determined to be influential (and hence estimated), we believe these reactions represent the sequential steps of fibrinopeptide a (FpA) and fibrinopeptide b (FpB) cleavage by thrombin, respectively. During fibrin polymerization, when thrombin binds fibrinogen, it cleaves FpA and FpB. This action exposes knobs ‘A’ and ‘B’ to which other fibrin monomers bind, thereby creating a fibrin (polymeric) matrix. In adult fibrinogen, FpA cleavage occurs at a faster rate than FpB cleavage [42]. Therefore, we predict that FpA cleavage is represented in equation (2.1), where fibrinogen is bound to thrombin and initially activated, and that FpB cleavage occurs in equation (2.2) where activated fibrinogen binds to additional thrombin. However, we note that this interpretation is limited owing to the inability of our absorbance-based data measurements to delineate fibrin monomers from matrix. As outlined in §3.2, removal of equation (2.3) adversely impacted our ability to fit the data. Studies using different experimental modalities to capture fibrin matrix formation, such as microscopy based techniques, will be considered in the future to enable the development of an extended model.

Regarding estimated values of influential parameters, we observe that low levels of sialic acid on adult fibrinogen resulted in a higher $k^{+}$ than unmodified fibrinogen, but medium levels of sialic acid on adult fibrinogen resulted in a lower $k^{+}$. This could indicate that small amounts of sialic acid on adult fibrinogen make fibrinogen’s binding regions more available to thrombin, but excess sialic acid addition could potentially block or hinder access to these binding sites. For the parameter $k_{1}^{+}$, we saw a positive trend where the adult fibrinogen with the highest amount of sialic acid had the highest $k_{1}^{+}$. Given our hypothesis that this parameter is associated with FpB cleavage, we believe sialic acid is mechanistically increasing thrombin’s ability to cleave FpB. This result is in line with our previous work which showed that fetal fibrinogen, a fibrinogen variant that exhibits elevated levels of sialic acid, had increased B-knob interactions compared to normal adult fibrinogen [32].

Understanding sialic acid’s impact on fibrinogen provides insight into how post-translational modifications influence protein structure and function. Altering post-translational modifications on proteins is an emerging method to improve therapeutic half-life, stability and efficacy [43,44]. Using these same principles, we can leverage sialic acid on fibrinogen to create finely tuned haemostatic agents.

The methodological approach presented in this study extends the parameter subset selection approach developed in our prior study [25] (and also applied to a different application in [45]) by incorporating randomization into local sensitivity-based parameter identifiability analysis. The inclusion of randomization facilitates a more comprehensive analysis of parameter candidates for model reduction, which is important when data are available for only one response variable. In particular, to determine candidate unidentifiable parameter subsets, many realizations were generated in step D and then analysed in step F for the model reduction (table 3). Similarly, many candidate identifiable parameter subsets were also generated to determine consistent subsets of influential parameters prior to parameter estimation (table 6). By contrast, our prior work did not employ randomization, effectively using one set of initial parameter values in the iterative parameter subset selection procedure. Here, our overall procedure yielded a reduced model with a consistent set of influential parameters that accurately fit the data across our three experimental designs, thus enabling a robust comparison of estimated values of influential parameters. While we used a similar randomized initialization to obtain and screen parameter estimates for our reduced model, this step could be replaced with an optimization algorithm that uses multiple, typically uniformly randomized, starting points for initializing the (local) optimization algorithm. Moreover, as noted in our earlier work [25] our fundamental identifiability analysis procedure (step D in figure 2) yields a significant reduction in error relative to a more ad hoc curve-fitting approach that would terminate after one iteration (e.g. iteration 0 versus iteration 7 in table 2). The incorporation of randomization in our extended approach also expands the scope of a systematic analysis of errors across many candidate models and model reductions, enabling screening for quality of curve-fits to the data. By contrast, the one-at-a-time nature of profile likelihood approaches typically generates fewer parameter candidates, scaling up in a more costly manner if extended to explore many more candidates. Although model sloppiness approaches [27] are also based on a linear algebraic decomposition of sensitivity matrices, prior studies have not carried out a systematic workflow for model reduction and parameter estimation across designs that incorporates exploration across many values in the parameter landscape through the incorporation of randomization.

Since our overall approach is rooted in local sensitivity derivatives, it is important to note that the resulting reduced models are not unique nor are they guaranteed to be optimal. This limitation is balanced by the incorporation of randomization, which greatly amplifies exploration of unidentifiable parameter candidates, candidate reduced models and estimated parameter values in a parameter space of moderate dimension. We also emphasize that parameter identifiability analysis improves the performance and accuracy of Bayesian approaches for uncertainty quantification, for example, by restricting MCMC sampling to only the identifiable subset of parameters [18].

## Data Availability

The data used in this study and the MATLAB code used to implement our algorithm are accessible via the repository link: http://doi.org/10.5281/zenodo.21829804.
